# Supplementary material for: Effect of Dietary Laminaria digitata with Carbohydrases on Broiler Production Performance and Meat Quality, Lipid Profile, and Mineral Composition
Source: Animals (Basel). 2022 Apr 13;12(8):1007. doi: 10.3390/ani12081007 (PMC9025196; doi:10.3390/ani12081007)
Supplement: Supplementary file 1 [file animals-12-01007-s001.zip › Table S1.pdf]

Table S1. Loadings for the first two principal components in the thigh muscle.

Variables	Factor 1	Factor 2
10:0	0.01	-0.28
12:0	0.46	0.45
14:0	0.85	0.31
14:1c9	0.77	0.03
15:0	-0.04	0.31
16:0	0.61	0.67
16:1c7	0.64	0.27
16:1c9	0.79	0.14
17:0	-0.44	0.49
17:1c9	0.59	0.17
18:0	-0.58	0.66
18:1c9	0.62	0.56
18:1c11	-0.65	0.59
18:2n6	-0.24	-0.93
18:3n6	0.00	-0.61
18:2t9t12	0.66	-0.33
18:3n3	0.61	-0.68
18:4n3	-0.42	0.07
20:0	-0.33	0.46
20:1c11	0.30	0.60
20:2n6	-0.48	-0.15
20:3n6	0.35	-0.43
20:4n6	-0.84	-0.17
20:3n3	0.04	0.39
20:5n3	-0.65	-0.47
22:0	-0.54	0.51
22:1n-9	-0.17	0.69
22:2n-6	0.14	-0.36
22:5n-3	-0.81	-0.31
22:6n-3	-0.84	0.34
Total lipids	0.81	-0.13
Cholesterol	0.14	0.20
α-Tocopherol	0.16	0.02
γ-Tocopherol	0.30	-0.01
Chlorophyll a	-0.61	-0.10
Chlorophyll b	-0.51	-0.05
Carotenoids	-0.47	0.06
Sodium	0.18	-0.38
Potassium	0.08	0.06
Calcium	0.17	-0.12
Magnesium	-0.01	0.29
Phosphorous	-0.08	0.16
Sulphur	-0.06	-0.02

Copper	0.13	0.04
Zinc	0.11	-0.07
Manganese	-0.03	-0.24
Iron	-0.09	0.09
