# Supplementary figures and images for: Effect of Dietary Laminaria digitata with Carbohydrases on Broiler Production Performance and Meat Quality, Lipid Profile, and Mineral Composition
Source: Animals (Basel). 2022 Apr 13;12(8):1007. doi: 10.3390/ani12081007 (PMC9025196; doi:10.3390/ani12081007)

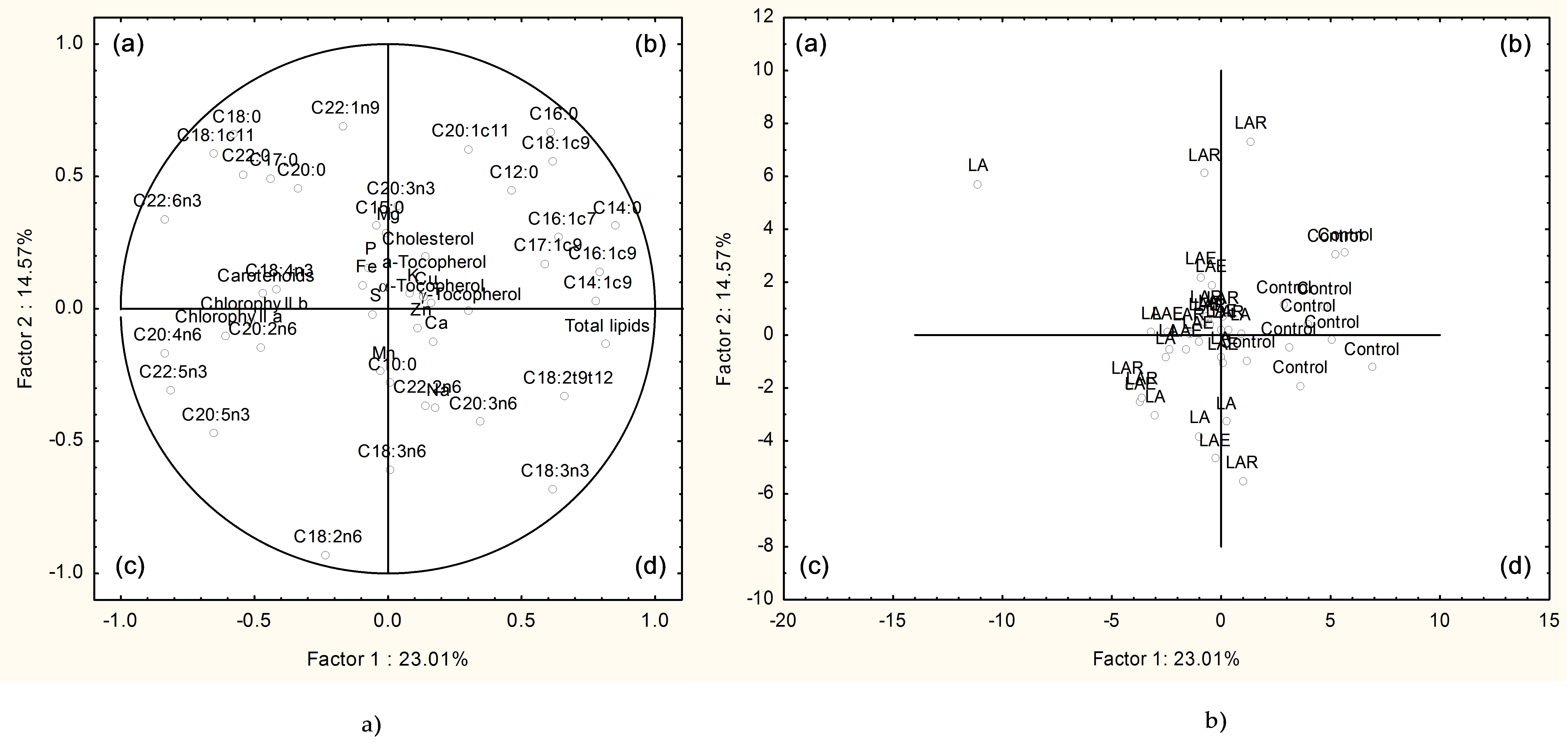

Supplement: Supplementary file 1 [file animals-12-01007-s001.zip › Figure S1.tif]
